# Oxygen Limitation Enhances the Antimicrobial Activity of Fosfomycin in *Pseudomonas aeruginosa* Following Overexpression of *glpT* Which Encodes Glycerol-3-Phosphate/Fosfomycin Symporter

**DOI:** 10.3389/fmicb.2018.01950

**Published:** 2018-08-21

**Authors:** Hidetada Hirakawa, Kumiko Kurabayashi, Koichi Tanimoto, Haruyoshi Tomita

**Affiliations:** ^1^Department of Bacteriology, Graduate School of Medicine, Gunma University, Maebashi, Japan; ^2^Laboratory of Bacterial Drug Resistance, Graduate School of Medicine, Gunma University, Maebashi, Japan

**Keywords:** antimicrobial resistance (AMR), multi-drug resistance (MDR), fosfomycin, anaerobiosis, cystic fibrosis

## Abstract

Fosfomycin is resurfacing as a “last resort drug” to treat infections caused by multidrug resistant pathogens. This drug has a remarkable benefit in that its activity increases under oxygen-limited conditions unlike other commonly used antimicrobials such as β-lactams, fluoroquinolones and aminoglycosides. Especially, utility of fosfomycin has being evaluated with particular interest to treat chronic biofilm infections caused by *Pseudomonas aeruginosa* because it often encounters anaerobic situations. Here, we showed that *P. aeruginosa* PAO1, commonly used in many laboratories, becomes more susceptible to fosfomycin when grown anaerobically, and studied on how fosfomycin increases its activity under anaerobic conditions. Results of transport assay and gene expression study indicated that PAO1 cells grown anaerobically exhibit a higher expression of *glpT* encoding a glycerol-3-phosphate transporter which is responsible for fosfomycin uptake, then lead to increased intracellular accumulation of the drug. Elevated expression of *glpT* in anaerobic cultures depended on ANR, a transcriptional regulator that is activated under anaerobic conditions. Purified ANR protein bound to the DNA fragment from *glpT* region upstream, suggesting it is an activator of *glpT* gene expression. We found that increased susceptibility to fosfomycin was also observed in a clinical isolate which has a promoted biofilm phenotype and its *glpT* and *anr* genes are highly conserved with those of PAO1. We conclude that increased antibacterial activity of fosfomycin to *P. aeruginosa* under anaerobic conditions is attributed to elevated expression of GlpT following activation of ANR, then leads to increased uptake of the drug.

## Introduction

Although fosfomycin is classified as an old antimicrobial agent, it has recently re-attracted. This drug is still effective against multidrug-resistant (MDR) pathogens because there is no structural relationship between the drug and other commonly used antimicrobials such as β-lactams including carbapenems, fluoroquinolones, and aminoglycosides (Cassir et al., 2014). Fosfomycin is transported into the bacterial cells via GlpT and UhpT, glycerol-3-phosphate and glucose-6-phosphate symporters, respectively, then inhibits MurA activity, which transfers phosphoenolpyruvate (PEP) to the 3′-hydroxyl group of UDP-*N*-acetylglucosamine in the initial step for bacterial cell wall biosynthesis (Kadner and Winkler, 1973; Argast et al., 1978; Bush, 2012). This drug is rather more effective under anaerobic conditions whereas most of other drugs decrease their activities (Inouye et al., 1989; Bryant et al., 1992; Morrissey and Smith, 1994; Grif et al., 2001; Kohanski et al., 2010). In our previous studies on *Escherichia coli*, we found that FNR (Fumarate Nitrate Reduction) and CRP (cAMP receptor protein) are positive regulators of both *glpT* and *uhpT* genes, and activities of these regulators increase under anaerobic conditions, which results in increased intracellular accumulation of fosfomycin (Kurabayashi et al., 2015b, 2017).

*Pseudomonas aeruginosa* is a well-studied opportunistic pathogen. Its serious infections often occur in not only compromised hosts but also patients suffering from chronic respiratory infections such as cystic fibrosis (CF) patients (Murray et al., 2007; Turner et al., 2014). Treatment of *P. aeruginosa* infections is generally difficult because of its innate tolerance to antimicrobial agents (Hoiby, 2011; Poole, 2011). In addition, advanced multidrug resistant (MDR) strains have been often isolated in clinical settings (Obritsch et al., 2004; Aloush et al., 2006). Recently, utility of fosfomycin is proposed for treating MDR *P. aeruginosa* infections although *P. aeruginosa* produces an enzyme from the chromosomally encoded *fosA* gene that inactivates fosfomycin by transferring glutathione molecule to this drug (Rigsby et al., 2004, 2005). *P. aeruginosa* is a facultative anaerobe, and it is able to grow with nitrate or nitrite when oxygen is restricted (Schreiber et al., 2007). During infections, this bacterium often encounters oxygen-limited situations, for instance, when it forms a poly-microbial structure as biofilm, where bacterial cells in biofilm compete with other bacterial members to utilize oxygen (Worlitzsch et al., 2002; Yoon et al., 2002). As well as *E. coli*, *P. aeruginosa* is more susceptible to fosfomycin under anaerobic conditions compared to when it is grown under aerobic conditions (Inouye et al., 1989). However, unlike *E. coli* and its related members, *P. aeruginosa* lacks *fnr* and *uhpT* genes while *glpT* gene is conserved, hence fosfomycin uptake depends on only GlpT (Stover et al., 2000; Castaneda-Garcia et al., 2009).

We have been interested in obtaining insights into the mechanism how fosfomycin is more effective against anaerobically grown cells, which enables us to create an idea to enhance efficacy of fosfomycin treatment. In this study, we aim to study the mechanism how *P. aeruginosa* is sensitive to fosfomycin in situations grown under anaerobic conditions.

## Materials and Methods

### Bacterial Strains and Culture Conditions

The bacterial strains and plasmids used in this study are listed in **Table 1**. A non-PAO1 *P. aeruginosa* clinical isolate designated Ps.a-682 was originally isolated from sputum in a hospitalized patient and is highly resistant to levofloxacin and aztreonam (MICs of 64 and 32 mg/L, respectively) and intermediate resistant to piperacillin and cefepime (MICs of 32 and 8 mg/L, respectively). Except in experiments to test drug susceptibility, all bacteria were grown in LB (Luria-Bertani) medium (Nacalai tesque, Kyoto, Japan). To grow *P. aeruginosa*, we supplied 0.5% (w/v) of potassium nitrate (KNO_3_) into the medium because this compound can be typically utilized as a substrate in the denitrification for facultative anaerobic growth (Schreiber et al., 2007). For drug susceptibility tests, bacteria were grown in Mueller Hinton medium (Becton Dickinson and Company, Franklin Lakes, NJ, United States) containing 0.5% of KNO_3_. *P. aeruginosa* strains were aerobically grown in glass tube with shaking at 160 rpm. For anaerobic cultures, we used a sealed container with gas generators, AnaeroPack-Anaero (Mitsubishi Gas Chemical Co., Inc., Tokyo, Japan). The cell growth was monitored by absorbance at 600 nm. For marker selection and maintaining plasmids, antibiotics were added to growth media at the following concentrations; 300 mg/L carbenicillin and 100 mg/L gentamicin for *P. aeruginosa*, 150 mg/L ampicillin, 15 mg/L chloramphenicol, and 20 mg/L gentamicin for *E. coli*.

**Table 1:** Strains and plasmids used in this study.

Strain or plasmid	Relevant genotype/phenotype	Reference
**Strains**		
PAO1	*P. aeruginosa* laboratory strain	Stover et al., 2000
PAO1ΔglpT	*glpT* mutant from PAO1	This work
Ps.a-682	*P. aeruginosa* clinical isolate from sputum	This work
MG1655	*E. coli* K12, wild-type, reporter strain	Blattner et al., 1997
MC4100	*E. coli* K12, lacking the endogenous *lacZ* gene	Casadaban, 1976
Rosetta^TM^(DE3)	T7-expression strain, Cm^R^	Novagen/EMD Bioscience
		
**Plasmids**		
pEX18Gm	Suicide vector with a *sacB* gene; Gm^R^	Hoang et al., 1998
pBBR1MCS4	Broad range host vector; Ap^R^	Kovach et al., 1995
pBBR1MCS4lacZ	pBBR1MCS4 with promoterless *lacZ*; Ap^R^	This work
pBBRglpT(PAO1)-P	*glpT* promoter reporter from PAO1; Ap^R^	This work
pBBRglpT(Ps.a-682)-P	*glpT* promoter reporter fromPs.a-682; Ap^R^	This work
pBBRfosA(PAO1)-P	*fosA* promoter reporter from PAO1; Ap^R^	This work
pBBRglpR(PAO1)-P	*glpR* promoter reporter from PAO1; Ap^R^	This work
pNN387	Single copy plasmid with promoterless *lacZ*; Cm^R^	Elledge and Davis, 1989
pNNglpT(PAO1)-P	*glpT* promoter reporter from PAO1; Cm^R^	This work
pBBR1MCS5	Broad range host vector; Gm^R^	Kovach et al., 1995
pBBR1MCS5glpT	GlpT expression; Gm^R^	This work
pTrc99A	Vector for IPTG-inducible expression; Ap^R^	Kurabayashi et al., 2015b
pTrc99Aanr	ANR expression in *E. coli*; Ap^R^	This work
pQE80L	Vector for expression of His-tagged protein; Ap^R^	Qiagen
pQE80anr	N-terminal His_6_-ANR overexpression plasmid; Ap^R^	This work

*Ap^R^, ampicillin resistance; Cm^R^, chloramphenicol resistance; Gm^R^, gentamicin resistance.*

### General Molecular Techniques

We used KOD-FX-Neo (TOYOBO, Osaka, Japan) as a DNA polymerase for PCR, and PCR was run on Bio-Rad T100 Thermal cycler (Bio-Rad, Hercules, CA, United States). The amplified DNA fragments were purified with PureLink PCR purification kit (Thermo Fisher Scientific, Waltham, MA, United States) when necessary. Restriction enzymes were purchased from New England Biolabs Inc. (Ipswich, MA, United States). We used Ligation high Ver.2 as a reagent containing DNA ligase (TOYOBO, Osaka, Japan). Plasmids were prepared from *E. coli* host using Wizard Plus SV Minipreps DNA Purification Systems (Promega Corp., Madison, WI, United States). These reagents and kits were used according to the manufacturer’s protocol. DNA sequencing was performed by the contract service supplied from Eurofins Genomics (Tokyo, Japan).

### Cloning and Mutant Constructions

To construct in-frame deletion of *glpT* in PAO1 background, we performed a gene replacement strategy based on homologous recombination using pEX18Gm vector as previously described (Hoang et al., 1998). We amplified a flanking DNA fragment including both upstream and downstream region of *glpT* by sequence overlap extension PCR with primer pairs, delta1/delta2 and delta3/delta4 primers as listed in **Table 2**. The upstream flanking DNA included 450 bp and the first three amino acid codons. The downstream flanking DNA included the last two amino acid codons, the stop codon, and 450 bp of DNA. These deletion constructs were ligated into BamHI and HindIII-digested pEX18Gm and introduced into PAO1. We selected sucrose-resistant/gentamicin-sensitive colonies, and confirmed the resulting mutant strains using PCR analysis and DNA sequencing.

**Table 2:** Primers used in this study.

Primer	DNA sequence (5′–3′)	Use
glpT-PA-delta1	gcgggatccgtcggcgtgttctggggatc	*glpT* deletion construction
glpT-PA-delta2	gagccgccgcgtcatcagccggctccgaacatcgcgagctccg	*glpT* deletion construction
glpT-PA-delta3	aagcggagctcgcgatgttcggagccggctgatgacgcggcgg	*glpT* deletion construction
glpT-PA-delta4	gcgaagcttatctggcggaactgcccgac	*glpT* deletion construction
lacZ-F	gcgggtacctttcacacaggaaacagctatg	pBBR1MCS4lacZ construction
lacZ-R	gcctctagattatttttgacaccagaccaac	pBBR1MCS4lacZ construction
glpT-PA-PF-NsiI	gcgatgcatcgccctcggccagcgcagg	pBBRglpT(PAO1)-P and pBBRglpT(Ps.a-682) construction
glpT-PA-PR-KpnI	gcgggtaccgcgagctccgcttgttgttg	pBBRglpT(PAO1)-P and pBBRglpT(Ps.a-682) construction
fosA-PF	gcgatgcatatggtcgaggtcgacgtgc	pBBRfosA(PAO1)-P construction
fosA-PR	gcgggtaccgggggctccttgcaagatg	pBBRfosA(PAO1)-P construction
glpR-PF	gcgatgcatagccggagtgcgacgagcc	pBBRglpR(PAO1)-P construction
glpR-PF	gcgggtaccgggttgttctcgtgctgcc	pBBRglpR(PAO1)-P construction
glpT-PA-PF-NotI	gcggcggccgccgccctcggccagcgcagg	pNNglpT(PAO1)-P construction
glpT-PA-PR-HindIII	gcgaagcttgcgagctccgcttgttgttg	pNNglpT(PAO1)-P construction
pBBRglpT-PA-F	gcgggtaccgcgggccggagcgatacac	pBBR1MCS5glpT construction
pBBRglpT-PA-R	gcgaagctttcagccggcttgctgcggc	pBBR1MCS5glpT construction
pTrcanr-F	gcgccatggccgaaaccatcaaggtgc	pTrc99Aanr construction
pTrcanr-R	gcgggatcctcagccttccagctggccgc	pTrc99Aanr construction
pQE80anr-F	gcgggatccgccgaaaccatcaaggtgcg	pQE80anr construction
pQE80anr-R	gcgaagctttcagccttccagctggccg	pQE80anr construction
glpT-PA-PR-6FAM	tcgcgagctccgcttgttg	DNase I footprinting analyses
glpT-PA-RACE1	cagccgttgatgaagagcagg	5′-RACE analysis
glpT-PA-RACE2	ggccgaacggcaggaagacc	5′-RACE analysis
glpT-PA-RACE3	tggcgatggccgacatcgcg	5′-RACE analysis


We constructed *lacZ* reporter plasmids to measure promoter activities of *glpT* in PAO1 and Ps.a-682, and *fosA* and *glpR* in PAO1, designated pBBRglpT(PAO1)-P, pBBRglpT(Ps.a-682)-P, pBBRfosA(PAO1)-P, and pBBRglpR(PAO1)-P, respectively. The *lacZ* gene PCR-amplified from *E. coli* MG1655 (Blattner et al., 1997) was ligated into the broad-host-rage vector, pBBR1MCS4 (Kovach et al., 1995) digested with KpnI and XbaI to initially generate pBBR1MCS4lacZ. We, respectively, PCR-amplified the 300 bp region upstream of *glpT* from PAO1, and Ps.a-682 and *fosA* and *glpR* from PAO1 with primers listed in **Table 2**. These DNA fragments were, respectively, ligated into pBBR1MCS4lacZ digested with NsiI and KpnI, then the region sequence containing *lac* promoter between NsiI and KpnI sites on the parent vector was replaced with that of *glpT*, *fosA* or *glpR* promoter.

We also constructed pNNglpT(PAO1)-P, a *lacZ* reporter plasmid to measure *glpT* promoter activity of PAO1 in *E. coli*, the heterologous host background. We PCR-amplified the 300 bp region upstream of this gene with primers, glpT-PA-PF-NotI and glpT-PA-PR-HindIII, and ligated it into NotI and HindIII-digested pNN387 plasmid with promoterless *lacZ* (Elledge and Davis, 1989).

To construct *glpT* expression plasmid, pBBR1MCS5glpT, we PCR-amplified the *glpT* gene and its 201 bp region upstream, then ligated into pBBR1MCS5 (Kovach et al., 1995) digested with KpnI and HindIII.

To construct ANR and His_6_-ANR expression plasmids, pTrc99Aanr and pQE80anr, respectively, the *anr* gene was PCR-amplified with the primer pair shown in **Table 2**. These products were ligated into pTrc99A plasmid digested with NcoI and BamHI and pQE80L plasmid (Qiagen, Valencia, CA, United States) digested with BamHI and HindIII. All constructs were confirmed by DNA sequencing.

### Fosfomycin Susceptibility Assays

MIC assays were performed by a serial agar dilution method with the standard method of the Clinical and Laboratory Standards Institute (CLSI) (Clinical and Laboratory Standards Institute, 2011). The MICs were determined as the lowest concentration at which growth was inhibited. We also performed disk assays using BD Sensi-Disc Fosfomycin 50 (Becton Dickinson and Company, Franklin Lakes, NJ, United States). The susceptibility to fosfomycin of *P. aeruginosa* strains was evaluated by the diameter (mm) of inhibitory zones on the Mueller Hinton agar culture. For these assays, bacteria were aerobically or anaerobically grown for 20 h (for aerobic cultures) or 22 h (for anaerobic cultures) in the Mueller Hinton medium containing KNO_3_ because the anaerobic growth was moderately slower than aerobic growth even when KNO_3_ is present.

### Fosfomycin Active Transport Assays

Assays to test fosfomycin accumulation in bacterial cells were conducted as previously described (Kurabayashi et al., 2014). Bacteria were grown in 20 ml of LB medium containing 0.5% of KNO_3_ to late-logarithmic phase and re-suspended in 1 ml of LB. This suspension was incubated for 60 min at 37°C in the presence of 2 mg of fosfomycin per ml, and then washed at three times with hypertonic buffer (10 mM Tris [pH 7.3], 0.5 mM MgCl_2_ and 150 mM NaCl) to remove the antibiotic. Cells were re-suspended in 0.5 ml of distilled water and plated on LB agar to determine the number of colony forming units (CFU)/ml. The bacteria re-suspension was boiled at 100°C for 3 min to release the fosfomycin. After centrifugation, the antibiotic concentration in the supernatant was determined by a diffusion disk assay. In this assay, sterilized assay disks (13 mm; Whatman, Florham Park, NJ, United States) were saturated with 0.1 ml of the supernatant and deposited onto LB agar plates overlaid with a 1:10 dilution of an overnight culture of *E. coli* MG1655 as a reporter strain (Blattner et al., 1997). Commercial fosfomycin was used to make a standard curve (Wako Pure Chemical Industries, Ltd., Osaka, Japan). Fosfomycin concentration in supernatants was quantified by the diameter (mm) of inhibitory zones on the LB agar culture and represented as ng per 10^7^ cells.

### Overexpression and Purification of His_6_-ANR

N-Terminal six histidine tagged ANR protein, His_6_-ANR was expressed in and purified from *E. coli* Rosetta^TM^ (DE3) (Novagen/EMD Bioscience, Philadelphia, PA, United States). Bacteria containing recombinant plasmid were grown at 37°C to an OD_600_ of 0.4 in LB, 0.5 mM IPTG (isopropyl-b-D-thiogalactopyranoside) was then added, and culture growth was continued for 3 h. Cells were harvested and disrupted in EzBactYeastCrusher containing 60 mg/L lysozyme (ATTO, Tokyo, Japan). The equal volume of lysis buffer (20 mM Tris [pH 7.9], 500 mM NaCl and 10% glycerol) was added to the cell lysate. After centrifugation, the resulting supernatant was mixed with Ni-NTA agarose (Qiagen, Valencia, CA, United States) for 1 h. After washed with 50 mM imidazol twice, His_6_-ANR was eluted with 200 mM imidazol. The purified protein was dialyzed (1-kDa cutoff) in buffer consisting of 50 mM Tris (pH 8.0). The protein was >95% pure as estimated by SDS-PAGE and Coomassie brilliant blue staining. Protein concentration was determined using the Bio-Rad protein assay (Bio-Rad, Hercules, CA, United States). The purified protein was incubated with 100 mM DTT (dithiothreitol) and 0.1 mM (NH_4_)_2_Fe(SO_4_)_2_ in 50 mM Tris (pH 8.0) for 4 h prior to footprinting experiment. ANR carries a [4Fe–4S] cluster in the active center as well as FNR that is produced by *E. coli* (Yoon et al., 2007). FNR loses its activity in the presence of oxygen because ferrous ions in [4Fe–4S] cluster are oxidized to ferric irons, then dimeric active form is dissociated (Green et al., 1996). That protein is able to form a dimer in the presence of high concentration of reducing agent like DTT even under aerobic conditions (Sharrocks et al., 1991). Therefore, we expected that activity of the purified ANR protein can be maintained in the presence of high concentration of DTT together with ferrous ions.

### DNase I Footprinting

DNase I footprinting was performed using a previously described non-radiochemical capillary electrophoresis method on an ABI PRISM genetic analyzer equipped with an ABI PRISM GeneScan (Hirakawa et al., 2005). The 6-carboxyfluorescein (6-FAM)-labeled (5′), 301-bp DNA fragments (starting at 300 bp upstream of the *glpT* start codon and ending 1 bp upstream of the *glpT* start codon) was generated by PCR amplification with primer pairs a 6-FAM labeled reverse primer (glpT-PA-PR-6FAM) and an unlabeled forward primer (glpT-PA-PF-NsiI). The DNA fragment (0.45 pmol) was mixed with purified ANR protein (0–30 pmol) in a 50 μl reaction mixture. After incubation for 20 min at room temperature, DNase I (2.5 units, Promega Corp., Madison, WI, United States) was added. After incubation for 60 s at room temperature, samples were purified for electrophoresis on the ABI PRISM genetic analyzer apparatus.

### Promoter Assays

*P. aeruginosa* strains carrying pBBRglpT(PAO1)-P, pBBRglpT(PA-682)-P, pBBRfosA(PAO1)-P or pBBRglpR (PAO1)-P, each LacZ reporter plasmid were aerobically or anaerobically grown overnight (∼16 h) at 37°C in LB medium containing 0.5% of KNO_3_. *E. coli* MC4100 (Casadaban, 1976) strains carrying pNNglpT(PAO1)-P, the LacZ reporter plasmid together with pTrc99A or pTrc99Aanr, the *anr* overexpression plasmid were anaerobically grown at 37°C in LB medium containing 0.1 mM of IPTG. Each cell culture of 0.5 ml was transferred into a 1.5-ml microtube, and 50 μl of chloroform was added to lyse the cells. After incubation for 5 min, the chemiluminescent signal in the supernatant was generated using a Tropix Galacto-Light Plus kit according to the manufacturer’s protocol (Thermo Fisher Scientific, Waltham, MA, United States). The β-galactosidase activity was determined as the signal value normalized to an OD_600_ of 1.

### 5′-RACE (Rapid Amplification of cDNA End) Analysis

5′-RACE was performed according to a method as described previously (Kurabayashi et al., 2015a). We isolated total RNA from the logarithmic phase PAO1 and synthesized cDNA with the unique primer glpT-PA-RACE1 (starting at 396 bp inside the *glpT*-coding region). To obtain a single RACE product, two-step PCR were performed by using an oligo(dT) primer attached to a 22-base anchor sequence at the 5′ end [primer oligo(dT)-Anchor] and primer glpT-PA-RACE2 (starting at 300 bp inside the *glpT*-coding region) for the first round, and an anchor primer without the oligo(dT) sequence (PCR anchor) and primer glpT-PA-RACE3 (starting at 217 bp inside the *glpT*-coding region) for the second round. The obtained 260–290-bp product was TA-cloned, and eight clones were sequenced.

### Statistical Analysis

*P*-value in each assay was determined by unpaired *t*-test and two-way ANOVA with the GraphPad Prism version6.00.

## Results

### *P. aeruginosa* PAO1 Strain Is More Susceptible to Fosfomycin When Anaerobically Grown

Initially, to confirm that the susceptibility of *P. aeruginosa* to fosfomycin indeed increases under anaerobic conditions, we determined the MICs by an agar dilution method for *P. aeruginosa* when aerobically or anaerobically grown. In this study, we used the PAO1 strain because it has been commonly used for *P. aeruginosa* studies in laboratory. PAO1 exhibited a relatively low susceptibility to fosfomycin when aerobically grown possibly due to the production of inactivation enzyme from the *fosA* gene in chromosome (MIC: 64 mg/L) (**Table 3**). On the other hand, when anaerobically grown, the bacterial cells showed an eightfold lower MIC compared to the cells when aerobically grown (MICs: 8 mg/L for anaerobically grown cells vs. 64 mg/L for aerobically grown cells) (**Table 3**). To confirm the result of the agar dilution assays, we also performed disk assays. We found that the inhibitory zone of growth formed by fosfomycin for cells when anaerobically grown was larger than that for cells when aerobically grown (diameters: 24 ± 2 mm for anaerobically grown cells vs. 15 ± 1 mm for aerobically grown cells). These results indicate that *P. aeruginosa* PAO1 strain is more susceptible to fosfomycin when anaerobically grown.

**Table 3:** Fosfomycin MICs of *P. aeruginosa* and its derivatives.

Strain	Fosfomycin MICs (mg/L)	
	Aerobic	Anaerobic
PAO1	64	8
PAO1ΔglpT	>1024	>1024
PAO1ΔglpT/pBBR1MCS5	>1024	>1024
PAO1ΔglpT/pBBR1MCS5glpT	8	4
Ps.a-682	64	16


### The *glpT* Gene Expression of *P. aeruginosa* Increases when Anaerobically Grown, Resulting in Increased Uptake of Fosfomycin

**FIGURE 1:**
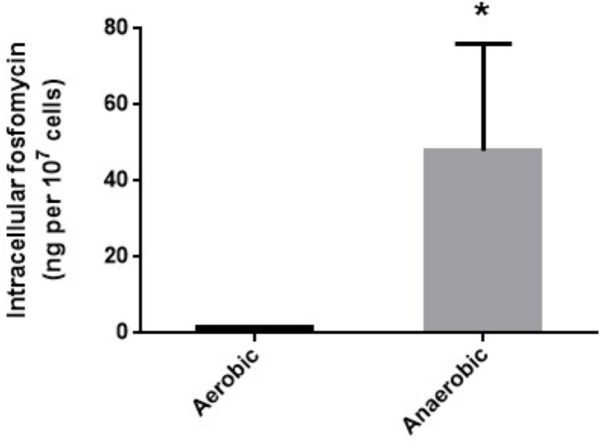
Intracellular accumulation of fosfomycin in PAO1 grown under aerobic or anaerobic conditions. Accumulation was described as amounts of fosfomycin (ng) in 10^7^ cells. Data plotted are the means from three independent experiments; error bars indicate the standard deviations, ^∗^*P*< 0.05. Asterisks denote significance for values of intracellular fosfomycin amount in bacterial cultures under anaerobic conditions relative to those under aerobic conditions.

We measured intracellular levels of fosfomycin in anaerobically and aerobically grown cells by transport assay as described in “Materials and Methods” section. The fosfomycin level in cells anaerobically grown was 28-fold higher than that in cells aerobically grown (1.7 ± 0.1 ng/10^7^ cells for aerobically grown cells vs. 47.9 ± 19.0 ng/10^7^ cells for anaerobically grown cells) (**Figure 1**).

**FIGURE 2:**
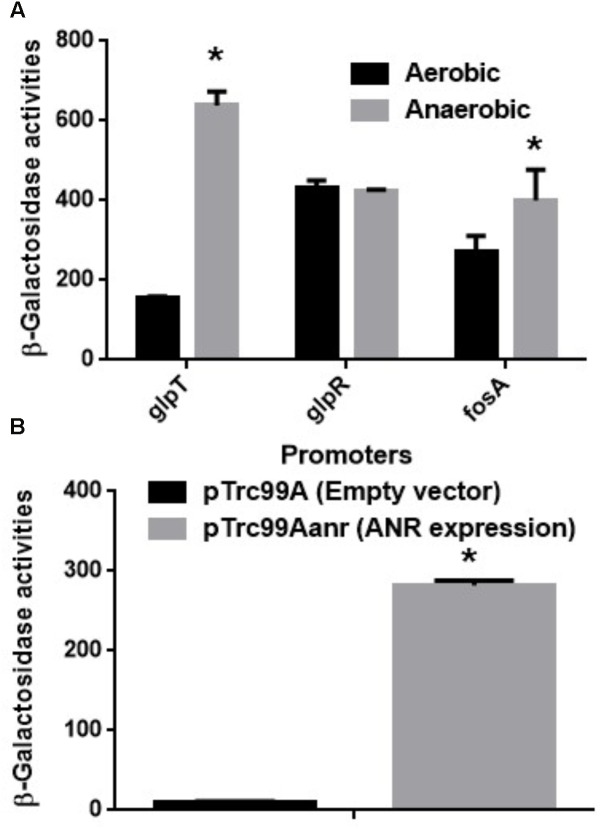
**(A)** β-Galactosidase activities of PAO1 containing pBBRglpT(PAO1)-P, pBBRglpR(PAO1)-P or pBBRfosA(PAO1)-P, the *lacZ* reporter plasmid grown under aerobic or anaerobic conditions. **(B)** β-Galactosidase activities correspond to *glpT* promoter activities of PAO1 in *E. coli* MC4100, the heterologous host containing pNNglpT(PAO1)-P, the *lacZ* reporter plasmid with pTrc99A (empty vector) or pTrc99Aanr (*anr* expression plasmid). Data plotted are the means from three independent experiments; error bars indicate the standard deviations, ^∗^*P*< 0.01. Asterisks denote significance for values of *glpT* promoter activity in PAO1 cultured under anaerobic conditions relative to those under aerobic conditions or in MC4100 with *anr* gene expression relative to those without *anr* gene expression.

GlpT is an only transporter for fosfomycin uptake in *P. aeruginosa* since it lacks the *uhpT* gene (Castaneda-Garcia et al., 2009). Therefore, we also measured promoter activity of *glpT* in PAO1 cells carrying the *lacZ* reporter plasmid when grown aerobically and anaerobically. The LacZ activity from the *glpT* promoter in anaerobically grown cells was 4.5-fold higher than that in aerobically grown cells (**Figure 2A**). In addition, we constructed the *glpT* mutant (designated as PAO1ΔglpT), then examined the susceptibility to fosfomycin when it was grown under aerobic and anaerobic conditions. PAO1ΔglpT was very highly resistant to this drug even when grown under anaerobic conditions (MICs: >1,024 mg/L; diameters of inhibitory zone: 7 ± 1 mm for both aerobic and anaerobic cultures) (**Table 3**). When pBBR1MCS5glpT as an exogenous *glpT* expression plasmid was introduced into this strain, its susceptibility was increased above the parent level because *glpT* could be additively expressed due to the leak from the *lac* promoter on the plasmid in addition to its own promoter (MICs: 4 mg/L for anaerobically grown cells vs. 8 mg/L for aerobically grown cells; diameters of inhibitory zone: 29 ± 1 mm for anaerobically grown cells vs. 23 ± 1 mm for aerobically grown cells) (**Table 3**). These combined results indicate that increased susceptibility of *P. aeruginosa* to fosfomycin under anaerobic conditions is due to elevated expression of GlpT that leads to increased intracellular accumulation of the drug.

GlpR is a repressor for the *glpT* gene expression, then it might participate in the increase in *glpT* expression during anaerobic growth. To test this hypothesis, we examined *glpR* expression between aerobic and anaerobic conditions by measuring its promoter activities in PAO1 carrying the *lacZ* reporter plasmid, pBBRglpR(PAO1)-P. However, we observed no significant difference in *glpR* expression between both conditions (**Figure 2A**). Apart from GlpT, *P. aeruginosa* produces chromosomally encoded FosA that confers innate tolerance by inactivating fosfomycin (Rigsby et al., 2004; Rigsby et al., 2005). As unexpected, the activity of *fosA* promoter in cells when grown anaerobically was rather slightly higher than that in cells when grown aerobically although we do not know this precise reason (**Figure 2A**).

### Anaerobic Regulator, ANR Contributes to Elevation of *glpT* Expression Under Anaerobic Conditions

ANR is a transcriptional regulator to activate subsets of genes that are essential for anaerobic growth in *P. aeruginosa* (Sawers, 1991). It is homologous with FNR of *E. coli*, and activated when oxygen is absent (Yoon et al., 2007). We hypothesized that ANR might contribute to elevation of *glpT* expression under anaerobic conditions. However, genes activated by ANR include *nar* operon which encodes nitrate reductase utilized for denitrification (Schreiber et al., 2007). Therefore deletion of *anr* notably decreases cell growth of *P. aeruginosa* when cultured under anaerobic conditions, then the growth defect might affect results of the promoter assay to evaluate *glpT* expression. To avoid this issue, we introduced pNNglpT(PAO1)-P, a *lacZ* reporter plasmid to measure *glpT* promoter activity of PAO1, together with pTrc99Aanr, an ANR expression plasmid or pTrc99A, the empty vector, into *E. coli*, the heterologous host lacking its chromosomal *lacZ* gene. We found that a LacZ expression from the reporter plasmid in *E. coli* carrying pTrc99Aanr was 28-fold higher than that in *E. coli* carrying the empty vector (**Figure 2B**).

**FIGURE 3:**
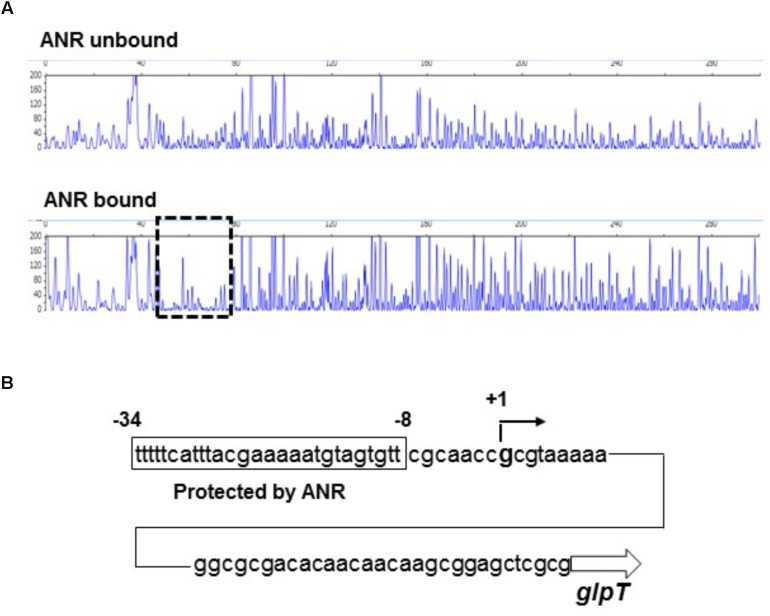
Sequence of the upstream *glpT* region and identification of ANR binding site and *glpT* transcriptional start site. **(A)** DNase I footprinting of the *glpT* promoter region. A 6-FAM-labeled DNA fragment was incubated in the presence or absence of ANR (30 pmol) and subjected to DNase I digestion. The fluorescence intensities of the DNA fragments (*y-*axis) are plotted relative to their size in bases (*x-*axis). One region (outlined by the dashed line) was protected from DNase I digestion in the presence of 30 pmol ANR protein. **(B)** Mapping of ANR binding site and *glpT* transcriptional start site on the *glpT* upstream region. The region protected from DNase I digestion is indicated by the box. Numbers indicate relative positions to the transcriptional start site.

We tested whether ANR binds to the region upstream of *glpT* including the promoter or not by DNase I footprinting analyses. As described in section “Materials and Methods,” to evaluate the activity of ANR to bind target DNA in the presence of oxygen, we incubated the ANR protein with high amount of DTT prior to the binding assay to reactivate the protein. We found that a 27-bp region located at 47- to 73-bp upstream of *glpT* translational start is protected from DNase I digestion by ANR (**Figure 3A**). The protected region contained the sequence (TTCAT-ttac-GAAAA) that is similar to a proposed ANR-binding consensus motif (TTGAT-N_4_-ATCAA) (Winteler and Haas, 1996; Yoon et al., 2007), suggesting that ANR binds to this site (**Figure 3B**). The result of 5’-RACE analysis showed that the transcription of *glpT* is initiated from a G residue located at 39 bases upstream of translational start site, which corresponds to 18 bases downstream of the proposed ANR-binding site (**Figure 3B**). These results indicate that ANR is an activator of *glpT* gene expression, and it contributes to elevation of GlpT expression during anaerobic growth of *P. aeruginosa*.

### Fosfomycin is More Active Under Anaerobic Conditions to not Only PAO1 but Also Another Clinical Isolate that Exhibits a Promoted Biofilm Phenotype

**FIGURE 4:**
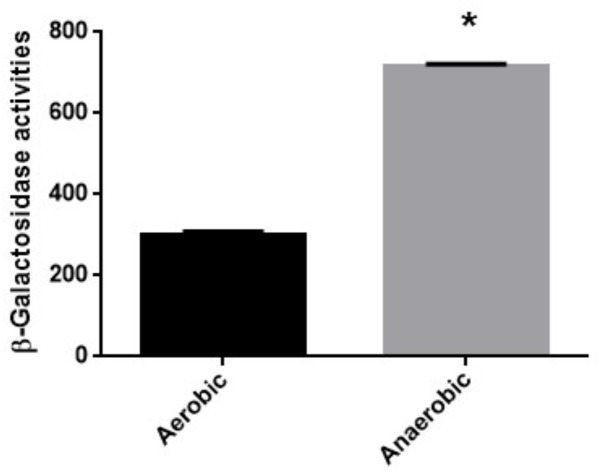
β-Galactosidase activities of Ps.a-682 containing pBBRglpT(Ps.a.-682)-P, the *lacZ* reporter plasmid grown under aerobic or anaerobic conditions. Data plotted are the means from three independent experiments; error bars indicate the standard deviations, ^∗^*P*< 0.01. Asterisks denote significance for values of *glpT* promoter activity in Ps.a-682 cultured under anaerobic conditions relative to those under aerobic conditions.

We also determined MICs of fosfomycin in a clinical isolate designated Ps.a-682 that highly forms biofilm when cultured under aerobic or anaerobic conditions since biofilm closely associates with bacterial virulence (Alhede et al., 2014). Therefore, we got interested in Ps.a-682 for this study. As well as PAO1, Ps.a-682 exhibited a fourfold lower MIC during anaerobic cultures compared to aerobic cultures (MICs: 16 mg/L for anaerobically grown cells vs. 64 mg/L for aerobically grown cells; diameters of inhibitory ring: 21 ± 2 mm for anaerobically grown cells vs. 15 ± 1 mm for aerobically grown cells) (**Table 3**). We also measured promoter activity of *glpT* by using the *lacZ* reporter plasmid, pBBRglpT(Ps.a-682)-P in Ps.a-682 when aerobically and anaerobically grown. A LacZ expression from the *glpT* promoter in cells grown anaerobically was higher than that in cells grown aerobically, which is similar to the results of the experiment with PAO1 (**Figure 4**). In addition, we determined sequences of the *anr* open reading frame and region upstream of *glpT* gene in Ps.a-682. These sequences were >90% identical to those of PAO1, then the region sequence in PAO1 protected from DNase I digestion including the (TTCAT-ttac-GAAAA) site where ANR is proposed to bind was completely conserved in Ps.a-682. These observations suggest that fosfomycin is more active under anaerobic conditions to not only PAO1 but also probably other *P. aeruginosa* strains, in which both *glpT* including its promoter and *anr* are conserved.

## Discussion

In our previous studies on *E. coli*, we showed that expression of both *glpT* and *uhpT* which encode transporters contributing to fosfomycin uptake is elevated via an activation of FNR, a global anaerobic regulator when anaerobically grown, which leads to increased susceptibility under anaerobic conditions (Kurabayashi et al., 2015b, 2017). In this study, we found that *P. aeruginosa* also becomes more susceptible to fosfomycin during anaerobic cultures as well as *E. coli* (**Table 3**), then expression of *glpT* and intracellular level of fosfomycin are elevated by following activation of ANR, an anaerobic regulator of *P. aeruginosa* (**Figures 1–4**) while *uhpT* and *fnr* are absent on *P. aeruginosa* chromosome (Stover et al., 2000; Castaneda-Garcia et al., 2009). Similar to FNR of *E. coli*, ANR carries a [4Fe–4S] cluster in the active center, and they form an active dimer in the absence of oxygen (Yoon et al., 2007). They bind to an identical DNA sequence (TTGAT-N_4_-ATCAA) although *glpT* including its region upstream in PAO1 has a low sequence identity (<50%) to that in *E. coli* (Winteler and Haas, 1996; Yoon et al., 2007). GlpR blocks the transcription of *glpT* while FosA is an enzyme to inactivate fosfomycin. We showed that these productions are unlikely affected by oxygen depletion (**Figure 2A**). Inactivation of *cyaA* and *ptsI* decreases susceptibility to fosfomycin in *E. coli*. However, we note that these gene products do not contribute to the susceptibility in *P. aeruginosa* (Castaneda-Garcia et al., 2009).

Glycerol-3-phosphate is a native substrate of GlpT transporter, and it is a sole carbon source for growth (Argast et al., 1978; Kurabayashi et al., 2014). In particular, *E. coli* is also able to use this compound as an electron donor for anaerobic respiration, where the electron is released in a conversion to dihydroxyacetone phosphate (DHAP) by anaerobic glycerol-3-phosphate dehydrogenase (GlpABC) and transferred to the terminal reductases via a menaquinone carrier (Varga and Weiner, 1995; Unden and Bongaerts, 1997). These literatures may convince us that *E coli* increases the storage of glycerol-3-phosphate by elevating GlpT production to sustain its fitness under anaerobic conditions. On the other hand, there is an idea that contribution of GlpT to fitness in *P. aeruginosa* is less than that in *E. coli* although metabolism involving glycerol-3-phosphate is relatively less studied in *P. aeruginosa* (Castaneda-Garcia et al., 2009). *P. aeruginosa* has a more versatile capability for carbon utilization than *E. coli*. Indeed, glycerol-3-phosphate supplement does not promote the cell growth when cultured even in minimal medium containing an alternative carbon source such as glucose or glycerol (Castaneda-Garcia et al., 2009). In addition, it is unlikely that it uses glycerol-3-phosphate for anaerobic respiration because it lacks menaquinone and GlpABC while glycerol-3-phosphate may be still able to be used for aerobic respiration involving aerobic glycerol-3-phosphate dehydrogenase encoded by *glpD* (Schweizer and Po, 1994). However, transcriptome studies showed that the expression of *glpD* is higher in primary strains isolated from CF patients than that of strains grown in laboratory medium, implying that GlpD might contribute to virulence of *P. aeruginosa* during chronic respiratory infections (Daniels et al., 2014; Son et al., 2007), which does not exclude a possibility that glycerol-3-phosphate metabolism associated with GlpT-mediated transport participates in the oxygen-limited lifestyle during infection.

The *in vitro* antimicrobial tests according to a guideline of the Clinical and Laboratory Standards Institute (CLSI) suggested that MICs of fosfomycin are very high for most of *P. aeruginosa* strains including clinical isolates (Clinical and Laboratory Standards Institute, 2011), suggesting its low efficacy to this bacterium. The *in vitro* antibacterial activity in common cases was evaluated in bacteria when cultured aerobically. However, *P. aeruginosa* is often under oxygen-depleted conditions during infection, for instance, when it forms a poly-microbial complex termed biofilm in patients suffering from chronic respiratory infectious diseases, such as CF (Singh et al., 2000). Therefore, data of *in vitro* CLSI test might underestimate the *in vivo* efficacy of fosfomycin. Some of *in vitro* studies showed that most of antimicrobials including tobramycin and ciprofloxacin that are commonly used for treatment of *P. aeruginosa* infections are less effective under anaerobic conditions (Bryant et al., 1992; Morrissey and Smith, 1994; Kohanski et al., 2010), which is one of reasons why bacteria in biofilm are hard to be completely expelled. Thus, these literatures encourage us to use fosfomycin for treatment of chronic biofilm infection caused by *P. aeruginosa* although *P. aeruginosa* still produces glutathione-*S*-transferase to inactivate fosfomycin which is encoded by *fosA* on the chromosome.

Recently, a combination therapy using fosfomycin together with tobramycin, an aminoglycoside is being proposed for treatment of airway infections caused by bacteria including typically *P. aeruginosa* in CF patients (McCaughey et al., 2012; Diez-Aguilar et al., 2015). It is expected to confer a synergistic bactericidal activity to *P. aeruginosa* because fosfomycin addition reduced transcripts of *narG/H* genes which encode nitrate reductase subunits being essential for anaerobic nitrate respiration when grown with tobramycin although the molecular mechanism has still been unknown (McCaughey et al., 2013). This literature suggests that the co-drug treatment is effective to *P. aeruginosa* settled in biofilm sites probably under oxygen-depleted conditions by inhibiting the nitrate respiration in addition to the innate antimicrobial activity of these drugs such as inhibition of cell-wall and protein syntheses. In other works, administration of fosfomycin together with β-lactams such as imipenem is expected to synergistically increase the bactericidal activity to *P. aeruginosa* by blocking peptidoglycan (PG) recycling (Borisova et al., 2014; Hamou-Segarra et al., 2017).

Fosfomycin increasingly interests as an alternative option for treatment of infections, particularly caused by refractory strains as multi-drug resistant *P. aeruginosa* (MDRP) while a shortage of new antimicrobial agents becomes a critical issue at present. We believe that this study will contribute to provide extensive information to make fosfomycin treatment more effective to *P. aeruginosa* infection rising a serious health issue in worldwide.

## Author Contributions

All of the authors designed the research. HH and KK performed the research. HH, KK, KT, and HT analyzed the data; HH, KT, and HT wrote the paper.

## Conflict of Interest Statement

The authors declare that the research was conducted in the absence of any commercial or financial relationships that could be construed as a potential conflict of interest.
